# The Cayman Crab Fly Revisited — Phylogeny and Biology of *Drosophila endobranchia*

**DOI:** 10.1371/journal.pone.0001942

**Published:** 2008-04-09

**Authors:** Marcus C. Stensmyr, Regina Stieber, Bill S. Hansson

**Affiliations:** Department of Evolutionary Neuroethology, Max Planck Institute for Chemical Ecology, Jena, Germany; The Rockefeller University, United States of America

## Abstract

**Background:**

The majority of all known drosophilid flies feed on microbes. The wide spread of microorganisms consequently mean that drosophilids also can be found on a broad range of substrates. One of the more peculiar types of habitat is shown by three species of flies that have colonized land crabs. In spite of their intriguing lifestyle, the crab flies have remained poorly studied. Perhaps the least investigated of the three crab flies is the Cayman Island endemic *Drosophila endobranchia*. Apart from its life cycle very little is known about this species, including its phylogenetic position, which has remained unresolved due to a cryptic set of characteristics.

**Principal Findings:**

Based on molecular data, corroborated by a re-analysis of the morphological make up, we have resolved the phylogenetic position of *D. endobranchia* and show that it somewhat surprisingly belongs to the large Neotropical *repleta* radiation, and should be considered as an aberrant member of the *canalinea* species group. Furthermore we also provide additional data on the behavior of these remarkable flies.

**Conclusion:**

Our findings reveal that the two Caribbean crab flies are not as distantly related as first thought, as both species are members of the derived *repleta* radiation. That this lineage has given rise to two species with the same odd type of breeding substrate is curious and prompts the question of what aspects of their shared ancestry has made these flies suitable for a life on (and inside) land crabs. Knowledge of the phylogenetic position of *D. endobranchia* will allow for comparative explorations and will aid in efforts aimed at understanding processes involved in drastic host shifts and extreme specialization.

## Introduction

**Figure 1:**
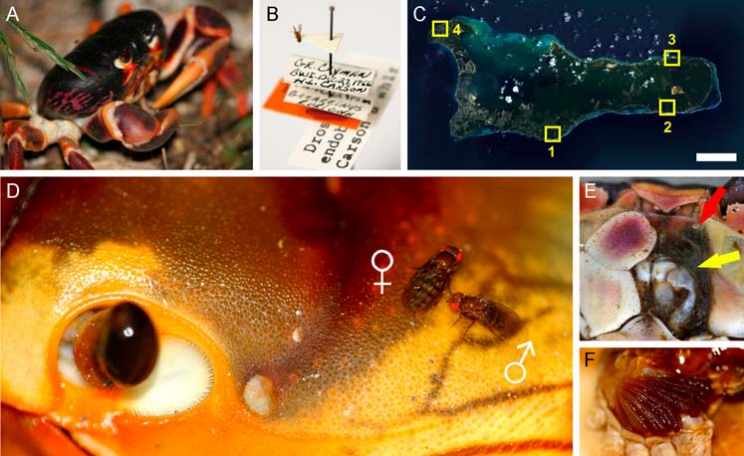
The “crabitat”. (A) The black crab (*Gecarcinus ruricola*, black morph). (B) The male holotype of *D. endobranchia* collected by H.L. Carson in 1966, now in the collections of the National Museum of Natural History, Washington, D.C. (C) Grand Cayman; Numbers refer to sites were crab flies were found. Scale bar 5 km. Image courtesy of NASA. (D) Male fly courting a female fly under the watchful eye of their host (a yellow morph black crab). (E) First instar fly larvae are found in the nephric pads (yellow arrow). The larvae feed on microorganisms, which cleanse the urine (exuded from the green gland; red arrow) of nitrogenous waste compounds. (F) Second instar is spent inside the gill chambers.

Among drosophilid flies, what surely must be considered as one of the more outlandish types of habitat comes from three species of flies that have found a home on land crabs. An adaptation that seems to have arisen in a remarkable display of parallel evolution, as the three flies all stem from separate lineages and occur in distinct geographical localities. Two of the species are native to the Caribbean, whereas the third is exclusively found on Christmas Island in the Indian Ocean. The two Caribbean flies, *Drosophila carcinophila* and *D. endobranchia* live on Gecarnoid land crabs (Figure 1A), whereas the Christmas Island fly, *Lissocephala poweilli*, lives on both Brachyuran and Anomuran crabs (as e.g. the robber crab, *Birgus latro*). All three fly species complete their larval development on (and inside) their crab hosts, whereas the adult stage is to a varying degree associated with the crabs. *D. carcinophila* belongs to the large *repleta* species group (*mercatorum* subgroup) of the subgenus *Drosophila*, and is widespread throughout the Caribbean. *D. endobranchia* also belongs to the subgenus *Drosophila* but is unplaced as to species group, and is found only in the Cayman Islands (perhaps also in the Guantanamo province of Cuba). The Christmas Island fly belongs to the primitive genus *Lissocephala*, and is accordingly quite removed from the former two. For a more detailed account on the history and biology of these flies, see [1], [2].

The least investigated of the crab flies is *D. endobranchia*, which is solely known from 21 specimens (Figure 1B) collected on Grand Cayman (Figure 1C) in December 1966. This species has to our knowledge not been reported since. The adult flies (Figure 1D) appear to be closely associated with their crab hosts (the black crab, *Gecarcinus ruricola* and the red crab, *G. lateralis*). The eggs are deposited around the crab's eyes. Upon hatching, the larvae migrate to the nephric pads (Figure 1E) where the larvae feed on microbes that cleanse the urine (exuded from a pore at the base of the pad) from nitrogenous waste compounds before the fluid is reabsorbed by the crab. Starting second instar, the larvae migrate to the gill chambers (Figure 1F), where they evidently can stay for an extensive time period (up to several months). Beginning third instar, the larvae return to the mouth parts where they form a halo around the mouth opening. When feeding ends, the larvae fall to the ground and pupate [3]. Apart from these observations, preciously little is known about these flies, including their exact phylogenetic position.

We here report the rediscovery of the Cayman Crab flies. Based on molecular and morphological data, we show that *D. endobranchia* is a member of the Neotropical *canalinea* species group (within the *repleta* radiation, sensu Throckmorton [4]) and, contrary to what was first assumed, actually quite closely related to the other Caribbean crab fly, *D. carcinophila*. Furthermore, we also provide new insights into the biology of these remarkable flies.

## Results and Discussion

### Rediscovery

In February 2007 we mounted a search for *D. endobranchia* on Grand Cayman. Aided by Carson's meticulous field notes [5] we initially examined the sites where the original specimens were collected. Unfortunately, these sites now housed either hotels or condos and consequently, neither crabs nor flies were located. Expanding the search to other sites fitting Carson's description of the black crab's preferred habitat (coastal sea grape (*Coccoloba uvifera*) forests standing on “quite old limestone (…) where the rocks are sloughing off in horizontal fashion, giving it flat rocks” [5]) yielded a small number of diminutive crabs, but no sign of the flies. We instead found that the preferred habitat of the black crabs of Cayman is quite different from Carson's description. First of all, the crabs prefer Karst limestone formations, which are anything but horizontal and where the eroded rocks offer numerous hiding places for the crabs. Second, the crabs prefer the inland forest to the coastal sea grape bush land. Accordingly, we also examined crabs from the appropriate habitat for signs of fly infestations. Suitable sites were scouted during day, and revisited after nightfall (as the crabs are nocturnal). This search strategy proved successful. On the 16th of January 2007 the flies were relocated in a forested area along Beach bay road (Figure 1C (site 1)). During the following 10 days we located three other sites (all with the same type of habitat), which also held fly infested crab populations (Figure 1C (sites 2–4)). All in all, we managed to collect 66 fly specimens.

### Fly biology

The flies strike a peculiar sight in real life. They essentially hardly move at all, are extremely reluctant in leaving their host crabs and are hard pushed to take flight. Although the flies are sluggish, the crabs on which they reside are anything but. Chasing after crabs through a pitch-black jungle (growing on a razor sharp labyrinthine limestone ground), while trying to aspirate flies from their carapaces is not trivial. Obtaining large amounts of flies in this way is simply a nightmare. The scarcity of the flies, and the nocturnal and shy nature of their hosts made it a daunting task to figure out the biology of these odd flies.

We know that courting and mating takes place on the crabs, as we noted these behaviors on a number of occasions. The males are clearly territorial and defend their “crabitats” from invaders; evident from the frequently observed male-male disputes. We did however never see any flies actually feeding on (or from) the crabs. The flies were typically found scurrying (or more often just resting) on the frontal part of the carapace, and to some extent on the frontal leg pairs. The position of 103 flies on a schematic crab is shown in figure S1A. Infected crabs were found to house between 1–6 flies (on average 1.6 flies/infected crab). Although, flies can live on both red crabs and black crabs, they seem to prefer the latter. We found very few red crabs, though Carson examined 73 specimens and found that 30% carried eggs, compared with 61% of the black crabs [3]. A distinction between the behavior of the red and black crabs is that the latter do not typically excavate its own burrows, but rather use evacuated burrows from other crab species (e.g. *Cardisoma guanhumi*) or preferably natural crevices, a difference that could potentially explain the flies' preference. The excavation process would pose a problem for the adult flies, which as stated most unwillingly leave their hosts, and would also increase the chance of the eggs being rubbed off.

Interestingly, male flies were overrepresented in our collections (constituting 75.0% of the total catch) as well as in Carson's (90.5%) [5]. Where are the females? Possibly, females might solely visit crabs for mating and egg-laying but not for feeding and resting, whereby we would have missed them, as we were unable to locate any flies off crabs. Alternatively, the females could be frequently switching host crabs. As females would spend proportionally more time traveling from crab to crab we would as a result also catch fewer females. Yet again, the observed ratio may accurately reflect the actual sex ratio of the population. Skewed sex ratios are known from a number of drosophilids, however, in all cases does the females outnumber the males [e.g. 6], [7]. Further work is needed to resolve this issue.

The flies locate their hosts using olfactory cues, as shown by the following experiment. Black crabs (from site 1 (Figure 1C)) were caught and individually placed in plastic Tupperware™ boxes with perforated lids, which were then placed on the forest floor of site 1 at dusk (paired with empty control boxes) and then examined at dawn. We used 15 crabs (any fly guests were previously removed) as baits in four independent experiments. The crabs attracted in total 8 flies (7 males; 1 female), control boxes none. What volatile compounds emitted from the crabs might the flies use? Head space sampling and subsequent gas chromatography linked mass spectroscopy analysis revealed extremely low levels of odor compounds in the collected samples (data not shown). Unluckily, no flies survived to allow linked gas chromatography-electrophysiology in the laboratory. We can thus not exclude that very low levels of crab-specific odors act as attractants for the flies. Another possibility is that the flies rely primarily on CO_2_ to locate their hosts, similar to e.g. mosquitoes [8]. However, in *D. melanogaster*, CO_2_ has been shown to be a potent repellent [9]. It would be interesting to know if *D. endobranchia*, in contrast to *D. melanogaster* and probably most other drosophilids, finds CO_2_ attractive. Experiments to test CO_2_ attractivity will be performed in the future. At present we can only conclude that the crabs emit an attractive odor, the identity of which still remains unknown.

Not all crabs are suitable as hosts. In site 1 (Figure 1C), we found that 61.6% of the 232 examined black crabs carried flies. Interestingly, also site 2 showed a similar infection rate (61.1%, 18 examined crabs). Only a handful of crabs were found at sites 3 and 4, thus no reliable estimate of infection rate is possible. Surprisingly, also Carson found an infection rate of the black crabs of 61% [5]. What is wrong with the ∼40% crabs that do not house flies? Presence of flies does not appear to be correlated with crab color (the black crabs come in three different color morphs), as all color morphs showed similar levels of infection (Figure S1B). Neither did the size of the crabs seem to be a crucial factor (Figure S1C, D). The conserved ratio might simply reflect the equilibrium between the crab and fly populations. In other words, the uninfected crabs may only be a random sample of the crab population, which do not share a common trait that would make them unsuitable as hosts. It seems a bit peculiar though that this equilibrium would have remained stable for ∼40 years, in spite of the drastically changed Caymanian landscape and dramatic declines in crab population levels.

Habitat destruction and hunting pose a clear threat to the long term survival of the black crabs on Grand Cayman, and accordingly also to their fly guests. The decline is evident by comparing the results from Carson's fieldwork with ours. The beach habitats where Carson evidently found large numbers of crabs are today practically void of any crab life. Nowadays, black crabs are confined to isolated forest patches, which in most (if not all) cases lack any form of protection, as e.g. site 1, which at the time of writing is being largely cleared for development. Accordingly, it seems wise to consider both crabs and flies as vulnerable (if not directly threatened) until we have a better grasp on the actual population sizes. As collection of flies may actually have a measurable effect on the overall population, any future research effort into *D. endobranchia* has to be conducted with utmost care.

### Phylogeny and evolutionary history

Although distinctive, *D. endobranchia* is somewhat complicated to place in the drosophilid phylogenetic tree. It clearly belongs to the subgenus *Drosophila*, as indicated by e.g. the structure of the genitalia. Closer placement is, however, difficult as the fly displays a cryptic set of characteristics. The subgenus *Drosophila* is composed of three main radiations (or sections) [4], [10], [11]; (i) *virilis-repleta*, (ii) *immigrans-tripunctata* and (iii) the Hawaiian drosophilids. Because *D. endobranchia* exhibits similarities with species from the *immigrans-tripunctata* radiation as well as with species from the *virilis-repleta* radiation, *D. endobranchia* was placed basally in the subgenus *Drosophila*, prior to the split of these two lineages, but remained unplaced as to species group [3].

To resolve the phylogenetic position of *D. endobranchia* more closely we have here undertaken a molecular approach. We sequenced five loci with known potential to resolve taxonomic relationships. The loci we chose to examine were *COII*, *28S*, *Adh*, *amd* and *Ddc* (first mitochondrial, remaining four nuclear). These genes were chosen because (i) they have in previous studies yielded reliable phylogenies at different taxonomic levels and (ii) their wide use means that GenBank (http://www.ncbi.nlm.nih.gov/Genbank/index.html; accessed December 2007) also holds a large number of homologous reference sequences from a wide range of drosophilids. Since the morphological characters place *D. endobranchia* in the subgenus *Drosophila*, we predominantly chose to sample taxa from this branch. In total, we examined 249 sequences from 149 different species. A list of all sequences included in the analysis is found in the supplementary material (Table S1).

The nucleotide sequences from the five examined loci of *D. endobranchia* were aligned with their corresponding homologous counterparts. We subjected the individual aligned datasets to a substitution saturation test (as implemented in DAMBE) to assess the phylogenetic potential (plots shown in Figure S2). For *COII*, as well as for *28S* data, the transition/transversion ratio rapidly decreased with increasing genetic distance, a telltale sign of transitional saturation, indicating a potentially poor phylogenetic signal. For the nuclear genes, both transitions and transversions remained largely informative. Having concluded that at least parts of the dataset provided sufficient phylogenetic signal for any meaningful analysis, we next attempted to reconstruct the phylogenetic position of *D. endobranchia*. To avoid any method inflicted bias, we used three different approaches: Bayesian Inference (BI), Maximum Parsimony (MP) and Neighbor Joining (NJ).

Not wholly surprising did the *COII* (70 taxa; 642 total sites; 294 variable sites (vs); 239 parsimony informative sites (ps)) partition fail to produce a reliable phylogeny. We obtained essentially unresolved trees with overall node supports ≤50 (irrespective of method). Any firm conclusions as to the position of *D. endobranchia* were accordingly difficult to draw. The NJ tree is shown in the electronic supplementary material (Figure S3A).

**Figure 2:**
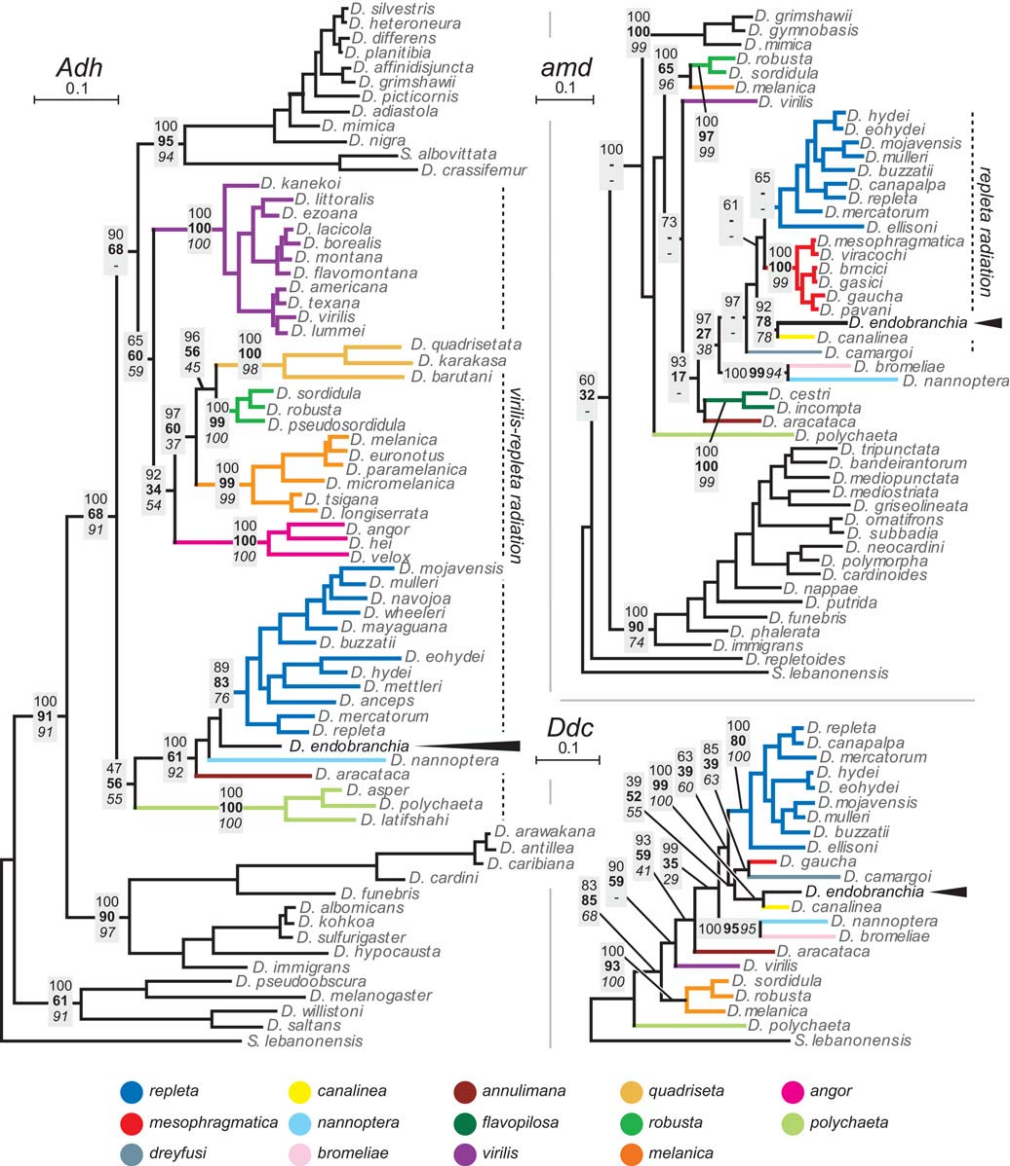
Phylogenetic trees constructed from the *Adh*, *amd* and *Ddc* partitions. Branch lengths and topology taken from the Bayesian inference trees (GTR+I+G model). Numbers refer to posterior probabilities obtained via Bayesian inference (BI, top; roman) and bootstrap support from Maximum Parsimony (MP, middle; bold) and Neighbor Joining (NJ, bottom; italic) analyses for the associated nodes. Support values are only shown for selected nodes. (-) indicates conflicting topologies obtained under MP and/or NJ compared with the shown BI topology. Alternate topologies are shown in the electronic supplementary material (Figure S4). Color coding refers to species groups included in the *virilis-repleta* radiation.

The nuclear data partitions did, however, perform better. The 71 taxa investigated for the *Adh* locus (652 total sites; 395 vs; 340 ps), yielded essentially similar phylogenies regardless of method (Figure 2). The main point of disagreement between the methods concerns the placement of the Hawaiian Drosophilidae, which under BI and MP clusters with the *virilis-repleta* radiation (as shown, and in accordance with [10]), and outside under NJ (alternate topology shown in Figure S4A). *D. endobranchia* clusters within the subgenus *Drosophila* with a high level of support (irrespective of method) and more precisely inside the *virilis-repleta* radiation (also with high support), close to the derived *repleta* species group (supported by all three methods, although with node support on the low side). The trees generated from the *amd* partition, containing 48 taxa (594 total sites; 302 vs; 268 ps) also support a placement of *D. endobranchia* inside the *virilis-repleta* radiation (Figure 2), more specifically within the large Neotropical *repleta* radiation that includes the *repleta*, *canalinea*, *dreyfusi*, *coffeata* and *mesophragmatica* species groups [4]. Moreover, the *amd* data proposes *D. endobranchia* as a sister-taxon to the *canalinea* group; a placement moderately to strongly supported by all three methods (GenBank holds no *Adh* sequences for any of the species of the *canalinea* group). The *amd* trees recovered with the different methods show some inconsistencies. The main incongruence (with a potential bearing on the placement to *D. endobranchia*) is the floating position of *D. ellisoni*, which in the Bayesian tree is placed basally in the *repleta* species group (as shown), whereas in the MP and NJ trees, *D. ellisoni* is found in a basal position relative to the sample species from the *repleta* radiation (Figure S4B, C). The position of *D. endobranchia* as a sister-taxon to *D. canalinea* is also supported by the phylogenies generated from the *Ddc* data partition. Trees obtained with the 22 taxa for *Ddc* (594 total sites; 302 vs; 268 ps) also points to an *endobranchia-canalinea* clade (with significant support) within the *repleta* radiation (Figure 2, alternate NJ topology in Figure S4D). The *28S* partition (38 taxa; 376 total sites; 133 vs; 70 ps) showed clear signs of saturation, and accordingly also turned out to have limited phylogenetic signal. The generated phylogenies were practically unresolved, with barely no nodes showing support above 50% level (irrespective of method). Even though the trees at large were unresolved, all methods grouped *D. endobranchia* with *D. canalinea* with high support (NJ tree is shown in Figure S3B).

**Figure 3:**
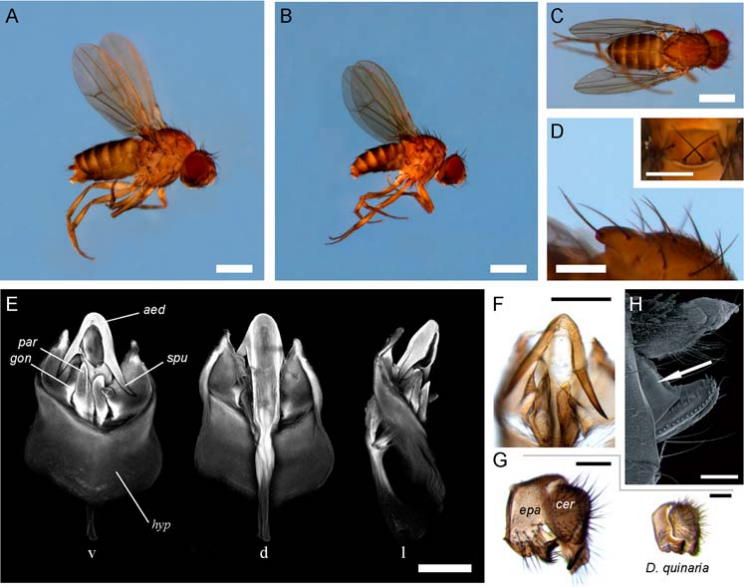
Morphological characteristics of *D. endobranchia*. (A) female and (B) male, left lateral view (C) Dorsal view of the female. Scale bars 1 mm. (D) Posterior scutellar setae are crossed (insert) and turned upright, which in combination with the erect dorsocentral setae give the flies their distinct bristly look. Scale bars 0.5 mm. (E) Male internal genitalia visualized by confocal laser scanning microscopy. Views from ventral (v), dorsal (d) and lateral (l) side. Aedeagus (aed), as well as hypandrium (hyp) display a close resemblance to the species of the *canalinea* group. Note the peculiar asymmetric shape of the spurs (spu, extending from the tip of the aedeagus), gonopod (gon) and paraphysis (par). (F) Stereomicroscope image of aforementioned characters. (G) The epandrium (epa) in *D. endobranchia* (left) is fused with the cerci (cer), a feature common in the *virilis-repleta* section, while rarely encountered in the *immigrans-tripunctata* section (here exemplified by *D. quinaria* (right, Tucson stock 15130–2011.00)). (H) Scanning electron micrograph of the female terminalia. The 8^th^ circumanal tergite (arrow) lacks the characteristic paragential fringe of the *canalinea* group. Scale bars in (E–H) equal 0.1 mm.

The molecular data thus strongly indicates a placement of *D. endobranchia* in the derived Neotropical *repleta* radiation, either as a new member of the *canalinea* group, or alternatively as a sole member of a novel species group, closely allied with the *canalinea* species. We next reexamined the morphological characteristics of *D. endobranchia* to resolve the placement vis-à-vis the *canalinea* group. Flies of both sexes were critically examined using a variety of microscopy techniques; a sample of some diagnostic characters is shown in Figure 3. A more complete redescription of *D. endobranchia* will be published at a later date. The analysis revealed a striking match of its male terminalia with those of the *canalinea* group. The shape of aedeagus, hypandrium and epandrium (Figure 3E–G) of *D. endobranchia* falls well within the variation displayed by the *canalinea* group (Figure S5). Furthermore, the flies' overall *bauplan* and pigmentation pattern (although much lighter overall) fits well with the *canalinea* group. However, some characters do not match. Most importantly, the 8^th^ circumanal tergite (Figure 3H) of the females does not show the distinct paragenital fringe displayed by all members of the *canalinea* group for which females are known. The paragenital fringe is however not unique to the *canalinea* group and is also found in *D. triangulina* (but not in the other species of the *tripunctata* group), and is possibly an adaptation to a specific breeding substrate shared by the *canalinea* group members (and perhaps also *D. triangulina*). The loss of this structure in *D. endobranchia* can accordingly be explained by its drastically different breeding substrate. Alternatively, the paragenital fringe might have been established only after the split of *D. endobranchia*. Why Carson and Wheeler failed to see the link with the *canalinea* flies is puzzling, especially since Wheeler himself proposed the group [12]. The reason might possibly be the missing paragential fringe, which at the time of *D. endobranchia*'s description (in 1968) was thought to be a unique and diagnostic feature of the *canalinea* group (as stated in [12]), as other species with this feature were yet to be reported (*D. triangulina* females were only properly examined in 1990 [13]). In conclusion, there seems to be no reason to invoke a novel species group for *D. endobranchia*, and accordingly we suggest that *D. endobranchia* should be placed as an aberrant member within the *canalinea* group, and taking a conservative approach, in its own subgroup.

What do we know about the *canalinea* group? Very little unfortunately. Of the 13 described species in the group, most are only known from their initial descriptions. The larval breeding sites of the group have been suggested to be dry fruits and blossoms [14], a view though which seems to be more of a qualified guess rather than based on actual observations. One of the species (*D. canalinioides*) has, however, been recorded on bracket fungus, which may indicate that the flies are fungus breeders. They are in any case very rare in standard fermenting fruit-baits, which would suggest a more specialized lifestyle. The scant records show that the group is forest dwelling and widespread throughout the Neotropics, with many species occurring in Central America and at least two in the Caribbean (Figure S6). The distribution pattern accordingly fits well with the notion of the species belonging to this group being the closest relatives of *D. endobranchia*. The finding of larvae from Guantanamo bay (Cuba) [3] displaying *D. endobranchia* characteristics is interesting, and points to a rather peculiar distribution pattern. Regrettably, repeated requests to the US naval command for permission to visit the Guantanamo base to confirm this observation has been met with silence. It would be interesting to know whether *D. endobranchia* occurs sympatrically in this area with *D. carcinophila*, a species with which it would directly compete.

The assessment of *D. endobranchia* having evolved prior to the split of the *immigrans-tripunctata* and *virilis-repleta* radiations thus seems to be nullified by the present results. Rather than being a relict, *D. endobranchia* is most likely the product of a more recent speciation event in the derived *repleta* radiation. The two Caribbean crab flies are consequently not as distantly related as first thought. It is intriguing that the *repleta* radiation has given rise to two species with the same odd habitat choice. What in their shared ancestry has made these flies suitable for a life on crabs would undoubtedly be interesting to know.

## Materials and Methods

### Flies, DNA and cloning

All fly specimens were collected on Grand Cayman and were aspirated directly from land crabs. Permission to collect flies was kindly granted by Cayman Islands Department of Environment. Flies destined for DNA and morphological analysis were stored in 100% ethanol. DNA was extracted according to standard protocols. Five gene regions, four nuclear and one mitochondrial, were amplified using PCR. The chosen mt locus was cytochrome oxidase subunit II (*COII*). Nuclear loci were: 28S ribosomal RNA (*28S*), alcohol dehydrogenase (*Adh*), alpha methyl dopa resistant protein (*amd*) and dopa decarboxylase (*Ddc*). Amplification primers were taken from the following publications: *COII* [15]; *Adh* [16], *amd* [11] and *Ddc* [17]. The *28S* primers were: (Forward) 5′CCCGAAGTATCC TGAATCTTTCG 3′ and (Reverse) 5′GCCCGATGAACCTGAATATCC 3′. PCR reactions were performed according to standard protocols, and the products directly sequenced (both directions) on a ABI 3730XL sequencer (Applied Biosystem) according to the manufacturer's protocol. When direct sequencing failed, the PCR fragments were cloned into a TOPO vector (Invitrogen) and the sequencing process outsourced to MWG Biotech AG. Multiple clones from each gene were sequenced. All sequences have been deposited with GenBank (accession numbers EU490429-EU490433).

### Phylogenetic reconstruction

Homologous *28S*, *COII*, *Adh*, *amd* and *Ddc* sequences from other drosophilids were downloaded from GenBank and aligned with the corresponding *D. endobranchia* sequences using ClustalX 1.83 with default parameters [18]. The resulting multiple alignments were examined and, if necessary, edited manually in BioEdit [19]. Potential saturation in the datasets was explored by plotting the number of substitutions versus the divergence (TN93) with the program DAMBE [20]. Multiple methods were used to reconstruct the phylogenetic position of *D. endobranchia*. Bayesian inference analysis was conducted in MrBayes-3.1.2 [21]. Appropriate substitution models for each of the investigated loci were estimated with the Akaike Information Criterion as implemented in Modeltest 3.7 [22]. Four chains were run simultaneously (three heated, one cold) for 2–4,000,000 generations (depending on the dataset), with tree space sampled every 100^th^ generation. The first 500,000–1,000,000 generations (again, depending on the dataset) were discarded as burn-in. The remaining trees were used to calculate strict consensus trees. Maximum Parsimony (MP) analysis was performed in PAUP*4.0b10 [23], with settings as follows; heuristic search, random addition (n = 100) of sequences, TBR branch swapping. Support level for tree nodes was assessed with a bootstrap analysis. Settings for the bootstrap calculations were: Heuristic search, random addition (n = 100) of sequence and 500 bootstrap replicates. In addition we also performed a Neighbor Joining (NJ) analysis using MEGA version 3.1 [24]. Nucleotide distances were estimated by the Kimura 2-parameter model. The reliability of the NJ trees was assessed with bootstrap tests (1000 replicates).

### Morphological analysis

Preparation of the internal male genitalia for Confocal Laser Scanning Microscopy (CLSM) followed largely [25]. Briefly, the terminal half of the male abdomen was cut and incubated for 2–2.5 h in 10% KOH at 72°C for soft tissue removal. After washing (2×) in 70% EtOh, the remaining cuticular exoskeleton tissue was placed in Gelmount (Sigma-Aldrich). The genitalia were dissected from the abdomen and the periphallic separated from the phallic structure. The phallic structure was subsequently visualized in a Zeiss LSM5 META CSLM and maximum intensity projection images compiled from the optical sections. The stereomicroscope images were taken with a Leica MZ16FA microscope equipped with a DFC420C camera. All images were subsequently adjusted in Photoshop (Adobe). The scanning electron microscope image of the female terminalia was generated following standard protocols.

## Supporting Information

Table S1 List of taxa included in the phylogenetic analysis and respective GenBank accession numbers of the five analyzed genes.
(0.05 MB PDF)

Figure S1 (A) Position of 103 flies on a schematic black crab. (B) Crabs (233 examined, site 1) of different color morph appears to be similarly attractive as fly hosts. (C) Number of fly eggs vs. carapace width. Data extracted from Carson's field notes from his 1966 field trip [5]. In Carson's data set larger crabs appear to be less attractive to flies, as well as crabs <25 mm. However, it should be noted that large crabs are underrepresented in Carson's dataset. (D) Data collected in 2007 indicate in contrast a positive correlation between carapace width and number of flies. However, given the low number of examined crabs, any firm conclusion as to the importance of size has to await further field work.
(0.62 MB PDF)

Figure S2 Transitions and transversions in the five analyzed genes plotted against distance.
(0.79 MB PDF)

Figure S3 Neighbor Joining trees generated from the COII (A) and the 28S (B) data partitions. Numbers indicate bootstrap support (1000 replications) for the corresponding node. Values <50% are not shown.
(0.57 MB PDF)

Figure S4 Alternate tree topologies based on Maximum Parsimony (MP) and Neighbor Joining (NJ) analysis of the Adh (A), amd (B, C) and Ddc (D) datasets. Numbers indicate bootstrap support for the corresponding node (1000 replications for NJ, 500 for MP). Values <50% are not shown.
(0.62 MB PDF)

Figure S5 Male internal genitalia from selected members of the canalinea group (redrawn from [13], [26], with kind permission from the publishers) compared with D. endobranchia (right). First and second row, aedeagus (ventral and lateral view respectively); third row, hypandrium (ventral view). Scale bar 0.1 mm.
(0.37 MB PDF)

Figure S6 Distribution record of the canalinea group (http://taxodros.unizh.ch and references [12]–[14], [27]–[34]). Satellite image courtesy NASA.
(0.40 MB PDF)
