# Supplementary material for: The Cayman Crab Fly Revisited — Phylogeny and Biology of Drosophila endobranchia
Source: PLoS One. 2008 Apr 9;3(4):e1942. doi: 10.1371/journal.pone.0001942 (PMC2275792; doi:10.1371/journal.pone.0001942)
Supplement: Table S1 — List of taxa included in the phylogenetic analysis and respective GenBank accession numbers of the five analyzed genes. (0.05 MB PDF) [file pone.0001942.s001.pdf]

Table S1.

Genus	Subgenus	Section	Group	Species	*COII*	*28S*	*Adh*	*Ddc*	*amd*
Drosophila	*Drosophila*	*immigrans - tripunctata*	*calloptera*	*D. atrata*	AY162972	-	-	-	-
				D. calloptera	AF478419	-	-	-	-
			cardini	*D. acutilabella*	AF519317	-	-	-	-
				D. antillea	-	-	AY695383	-	-
				D. arawakana	-	X71229	AY695384	-	-
				D. cardini	AF519319	AF184004	AY695386	-	-
				D. cardinoides	-	-	-	-	AY699258
				D. caribiana	-	-	AY695387	-	-
				D. neocardini	AY847770	-	-	-	AY699260
				D. polymorpha	-	-	-	-	AY699259
			funebris	*D. funebris*	AF478422	X71227	AB033643	-	AF293709
			guarani	*D. griseolineata*	-	-	-	-	AY699257
				D. guaru	AY847763	-	-	-	-
				D. maculifrons	AY847766	X71261	-	-	-
				D. ornatifrons	AY162978	X71259	-	-	AY699250
				D. subbadia	-	-	-	-	AY699251
			histrio	*D. histrio*	AF519322	-	-	-	-
				D. sternopleuralis	-	X71263	-	-	-
			immigrans	*D. albomicans*	-	-	AB033642	-	-
				D. hypocausta	-	-	AY044131	-	-
				D. immigrans	AF519324	X71231	M97638	-	AY699261
				D. kohkoa	-	-	AY044124	-	-
				D. rubida	-	X71257	-	-	-
				D. sulfurigaster	-	-	AY044127	-	-
			macroptera	*D. macroptera*	AF519329	AY081400	-	-	-
			pallidipennis	*D. pallidipennis*	AY162982	X71269	-	-	-
			quinaria	*D. curvispina*	AF519320	-	-	-	-
				D. falleni	-	X54952	-	-	-
				D. innubila	AF519325	-	-	-	-
				D. kuntzei	AF519326	-	-	-	-
				D. limbata	AF519327	-	-	-	-
				D. nigromaculata	AF519332	-	-	-	-
				D. occidentalis	AF519333	-	-	-	-
				D. phalerata	-	X71255	-	-	AF293721
				D. transversa	AF519342	-	-	-	-
				D. unispina	AF519344	-	-	-	-
			testacea	*D. orientacea*	AF519334	-	-	-	-
				D. putrida	AF519335	-	-	-	AF293723
				D. testacea	AF519341	-	-	-	-
			tripunctata	*D. bandeirantorum*	-	-	-	-	AY699256
				D. cuaso	AY162984	-	-	-	-
				D. mediopictoides	-	X71265	-	-	-
				D. nappae	-	-	-	-	AY699248

Continued

Genus	Subgenus	Section	Group	Species	*COII*	*28S*	*Adh*	*Ddc*	*amd*
Drosophila	*Drosophila*	*immigrans - tripunctata*	*tripunctata*	*D. mediopunctata*	-	AJ308085	-	-	AY699254
				D. mediostriata	-	-	-	-	AY699253
				D. paraguayensis	AY162987	-	-	-	-
				D. paramediostriata	AY162995	-	-	-	-
				D. tripunctata	AF519343	-	-	-	AF293728
		virilis-repleta	*angor*	*D. angor*	-	-	DQ471627	-	-
				D. hei	-	-	DQ471622	-	-
				D. velox	-	-	DQ471637	-	-
			annulimana	*D. Annulimana*	AY847756	-	-	-	-
				D. aracataca	-	X71241	AY750127	AF324965	AF324949
				D. gibberosa	-	X71243	-	-	-
				D. pseudotalamancana	-	X71271	-	-	-
			bromeliae	*D. bromeliae*	AF478418	X55278	-	AF324966	AF324950
			canalinea	*D. canalinea*	-	AF184011	-	AF324968	AF324952
				D. endobranchia	EU490433	EU490432	EU490429	EU490431	EU490430
			dreyfusi	*D. camargoi*	AF478421	X71245	-	AF324967	AF324951
			flavopilosa	*D. cestri*	AY847758	-	-	-	AY699246
				D. incompta	AY847764	-	-	-	AY699247
			melanica	*D. euronotus*	-	-	AY750126	-	-
				D. longiserrata	-	-	AY750118	-	-
				D. melanica	-	-	AY750123	AF324972	AF324956
				D. micromelanica	-	X71233	AY750124	-	-
				D. paramelanica	-	-	AY750125	-	-
				D. tsigana	-	-	AY750121	-	-
			mesophragmatica	*D. brncici*	AY847757	-	-	-	AY699238
				D. gasici	AY847762	-	-	-	AY699241
				D. gaucha	-	X71253	-	AF324971	AF324955
				D. mesophragmatica	AY847769	-	-	-	AY699243
				D. pavani	AY847771	-	-	-	AY699245
				D. viracochi	AY847773	-	-	-	AY699244
			nannoptera	*D. acanthoptera*	AF183968	-	-	-	-
				D. nannoptera	AF183971	AY081384	DQ471666	AF324975	AF324959
				D. pachea	AF183969	-	-	-	-
				D. wassermani	AF183970	-	-	-	-
			polychaeta	*D. asper*	-	-	DQ471619	-	-
				D. latifshai	-	-	DQ471634	-	-
				D. polychaeta	EF469556	X71225	AB033641	AF324976	AF324960
			quadriseta	*D. barutani*	-	-	DQ471642	-	-
				D. karakasa	-	-	DQ471649	-	-
				D. quadriseta	-	-	DQ471635	-	-
			repleta	*D. aldrichi*	-	AJ308081	-	-	-
				D. anceps	-	-	DQ471656	-	-
				D. arizonae	AY437291	AJ308078	-	-	-
				D. buzzatii	AF146169	X71247	M62743	AF324980	AF324947
				D. canapalpa	-	-	-	AF324981	AF324948

Continued

Genus	Subgenus	Section	Group	Species	*COII*	*28S*	*Adh*	*Ddc*	*amd*
Drosophila	*Drosophila*	*virilis-repleta*	*repleta*	*D. ellisoni*	DQ202012	-	-	AF324969	AF324953
				D. eohydei	-	-	DQ471659	AF324970	AF324954
				D. eremophila	DQ202013	-	-	-	-
				D. hexastigma	AF183967	-	-	-	-
				D. huaylasi	AY437299	-	-	-	-
				D. huichole	DQ202018	-	-	-	-
				D. hydei	DQ202020	X71249	DQ471660	AF293737	AF293712
				D. longicornis	DQ202024	-	-	-	-
				D. mainlandi	DQ202026	-	-	-	-
				D. mayaguana	AY437298	AJ308084	M62742	-	-
				D. mercatorum	DQ202028	-	DQ471664	AF324973	AF324957
				D. meridiana	DQ202029	-	-	-	-
				D. mettleri	-	-	M57300	-	-
				D. mojavensis	DQ202031	AJ308077	AY364501	genome	genome
				D. mulleri	DQ437711	AJ308083	X03048	AF324974	AF324958
				D. navojoa	DQ436089	AJ308079	AY156524	-	-
				D. neorepleta	DQ202033	-	-	-	-
				D. nigrodumosa	-	AJ308080	-	-	-
				D. nigrohydei	-	-	-	-	-
				D. nigrospiracula	DQ202034	-	-	-	-
				D. novemaristata	DQ437710	-	-	-	-
				D. pachuca	DQ202038	-	-	-	-
				D. parisiena	AY437296	-	-	-	-
				D. repleta	-	X71251	DQ471667	AF324977	AF324961
				D. ritae	DQ202042	-	-	-	-
				D. stalkeri	DQ202048	-	-	-	-
				D. wheeleri	-	AJ308082	M62851	-	-
			robusta	*D. pseudosordidula*	-	-	AY750137	-	-
				D. robusta	-	X71237	DQ471670	AF293747	AF293724
				D. sordidula	-	-	DQ471671	AF324979	AF324963
			tumiditarsus	*D. repletoides*	-	-	-	-	AF324962
			virilis	*D. americana*	-	-	DQ471655	-	-
				D. borealis	-	-	DQ471666	-	-
				D. ezoana	-	-	DQ471658	-	-
				D. flavomontana	-	-	DQ471668	-	-
				D. kanekoi	-	-	DQ471653	-	-
				D. lacicola	-	-	DQ471667	-	-
				D. littoralis	-	-	DQ471662	-	-
				D. lummei	-	-	DQ471663	-	-
				D. montana	DQ426799	-	DQ471665	-	-
				D. texana	-	-	DQ471669	-	-
				D. virilis	DQ426823	-	DQ471643	AF293749	AF293729

Continued

Genus	Subgenus	Section	Group	Species	*COII*	*28S*	*Adh*	*Ddc*	*amd*
Drosophila	*Drosophila*	Hawaiian -	*adiastola*	*D. adiastola*	-	-	M60791	-	-
		Drosophilidae	Fungus feeder	*D. nigra*	-	-	M60793	-	-
			grimshawi	*D. affinidisjuncta*	-	-	U63563	-	-
				D. grimshawi	-	-	genome	-	genome
				D. gymnobasis	-	-	-	-	AF293710
			Modified mouth parts	*D. mimica*	-	-	M60792	-	AF293716
			plantibia	*D. differens*	-	-	M63303	-	-
				D. heteroneura	-	-	M63287	-	-
				D. picticornis	-	-	M63392	-	-
				D. planitibia	-	-	M63390	-	-
				D. silvestris	-	-	M63291	-	-
	Engiscaptomyza		*crassifemur*	*D. crassifemur*	-	-	M60790	-	-
	Dorsilopha			*D. busckii*	AF519347	X71273	-	-	-
	Sophophora		*melanogaster*	*D. melanogaster*	-	-	genome	-	-
			saltans	*D. saltans*	-	-	genome	-	-
			willistoni	*D. willistoni*	-	-	U95266	-	-
			obscura	*D. pseudoobscura*	-	-	genome	-	-
Scaptomyza	*Tantalia*	*		*S. albovittata*	-	-	M80925	-	-
Scaptodrosophila			*victoria*	*S. lebanonensis*	-	-	X53429	AF293739	AF293714

* *Hawaiian Drosophilid*
