# Supplementary figures and images for: The Cayman Crab Fly Revisited — Phylogeny and Biology of Drosophila endobranchia
Source: PLoS One. 2008 Apr 9;3(4):e1942. doi: 10.1371/journal.pone.0001942 (PMC2275792; doi:10.1371/journal.pone.0001942)

Figure S1

A

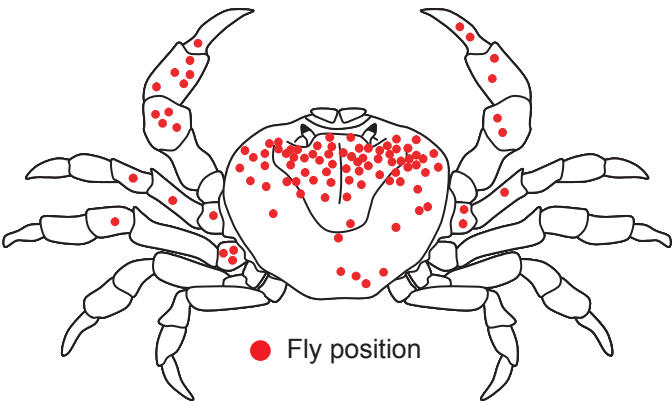

B

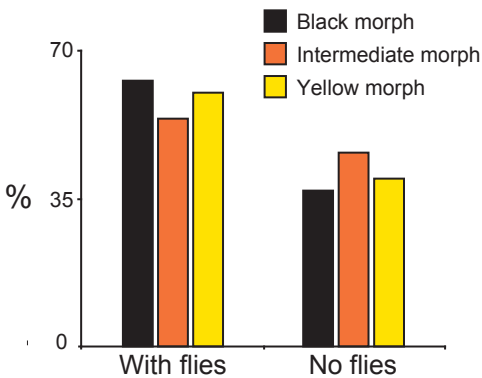

C

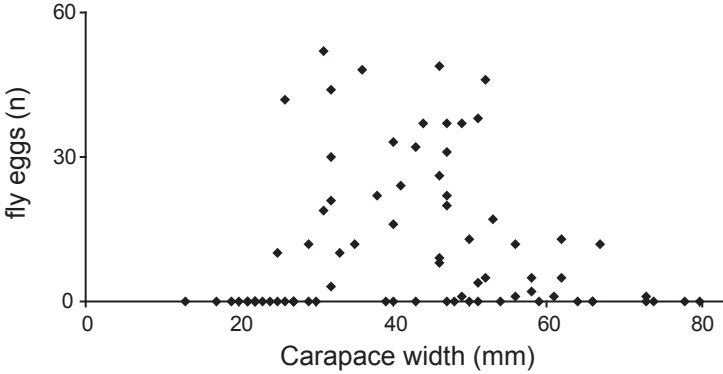

D

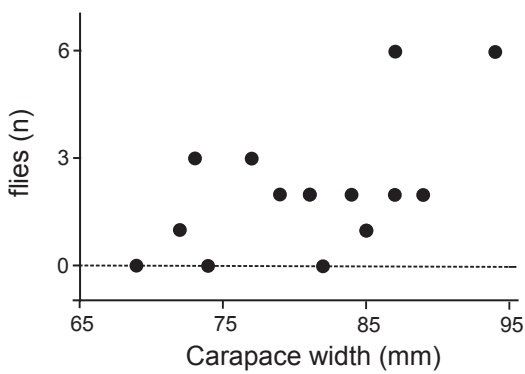

Supplement: Figure S1 — (A) Position of 103 flies on a schematic black crab. (B) Crabs (233 examined, site 1) of different color morph appears to be similarly attractive as fly hosts. (C) Number of fly eggs vs. carapace width. Data extracted from Carson's field notes from his 1966 field trip [5]. In Carson's data set larger crabs appear to be less attractive to flies, as well as crabs <25 mm. However, it should be noted that large crabs are underrepresented in Carson's dataset. (D) Data collected in 2007 indicate in contrast a positive correlation between carapace width and number of flies. However, given the low number of examined crabs, any firm conclusion as to the importance of size has to await further field work. (0.62 MB PDF) [file pone.0001942.s002.pdf]

Figure S2

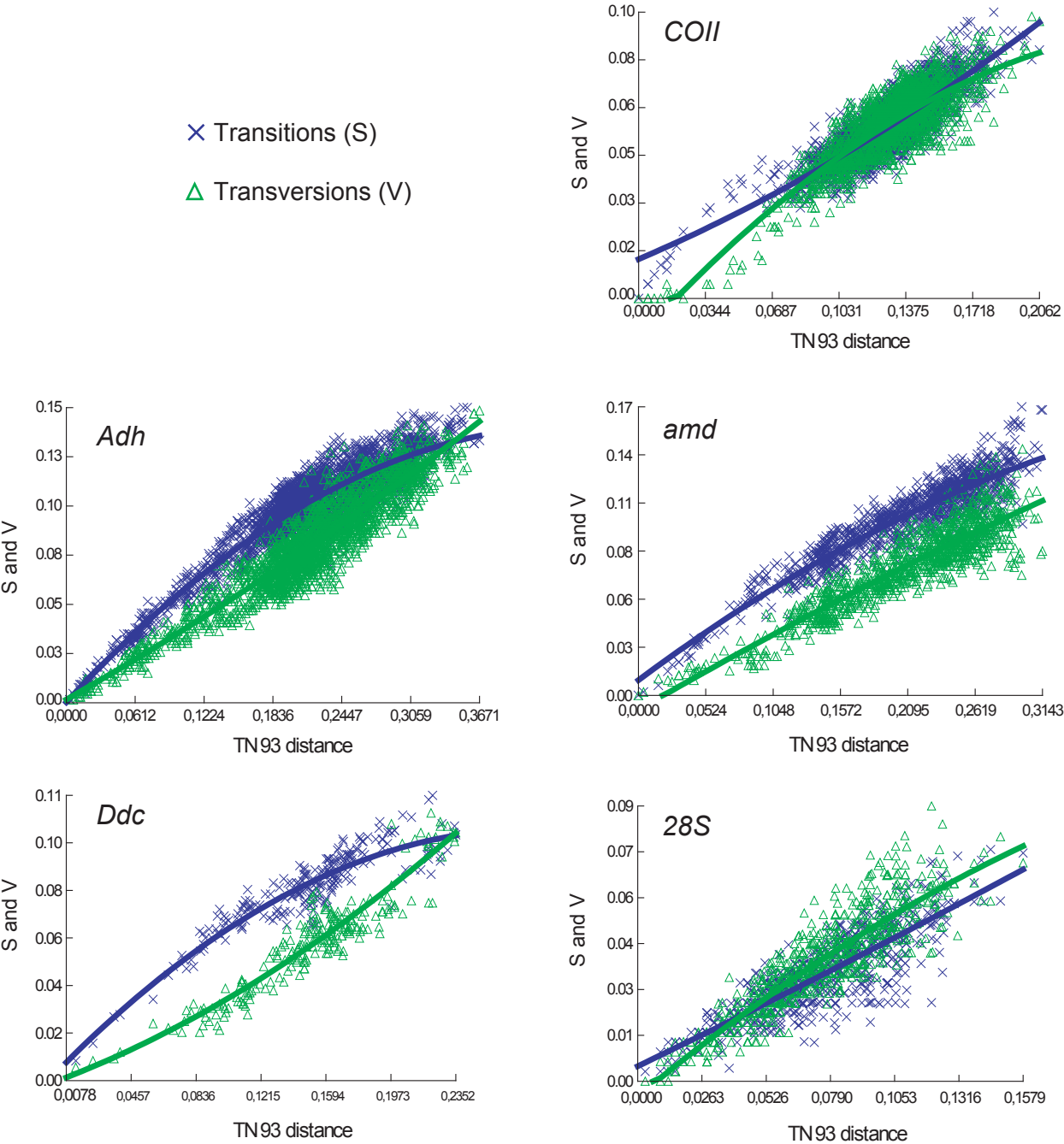

Supplement: Figure S2 — Transitions and transversions in the five analyzed genes plotted against distance. (0.79 MB PDF) [file pone.0001942.s003.pdf]

**Figure S3**

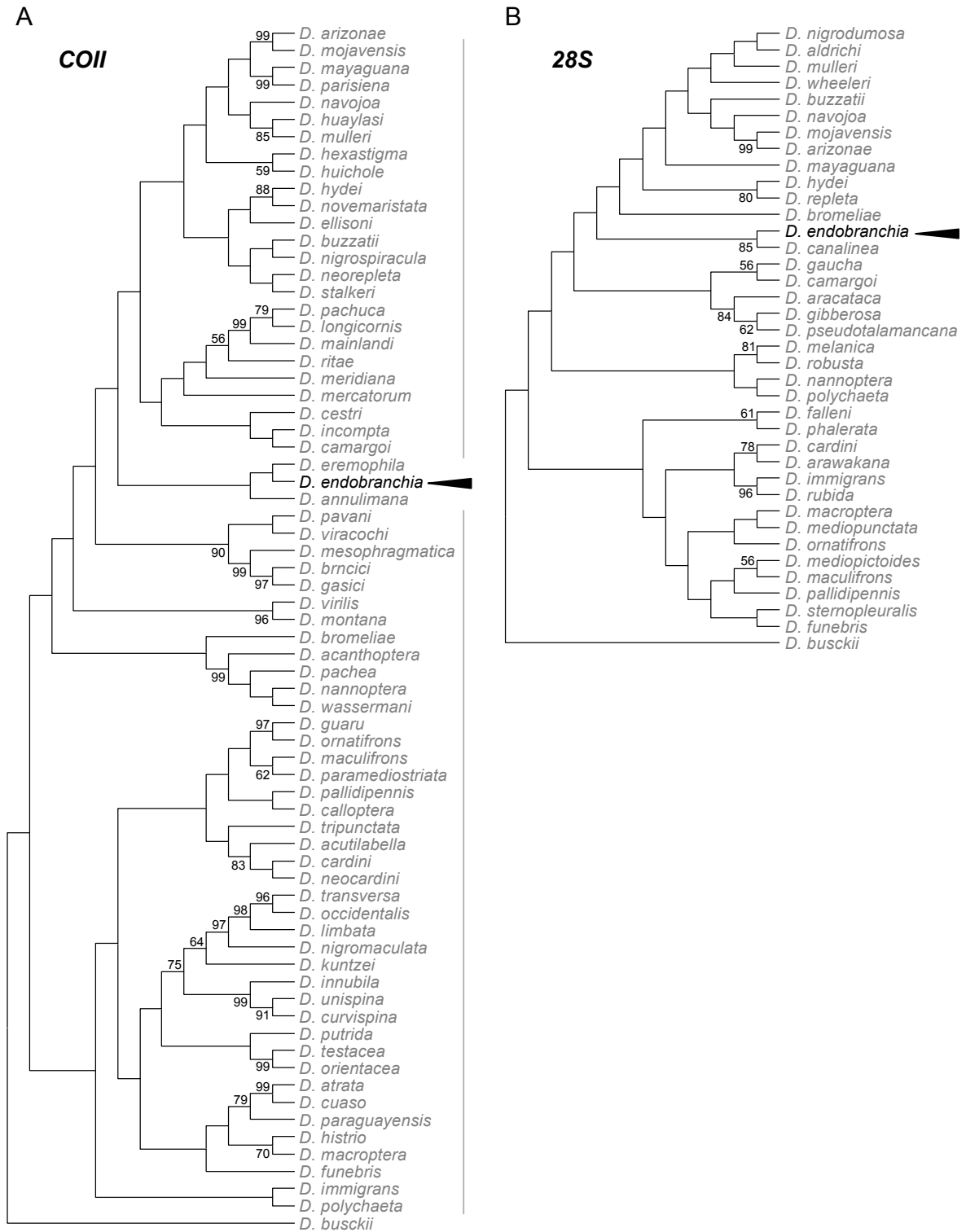

Supplement: Figure S3 — Neighbor Joining trees generated from the COII (A) and the 28S (B) data partitions. Numbers indicate bootstrap support (1000 replications) for the corresponding node. Values <50% are not shown. (0.57 MB PDF) [file pone.0001942.s004.pdf]

Figure S4

A

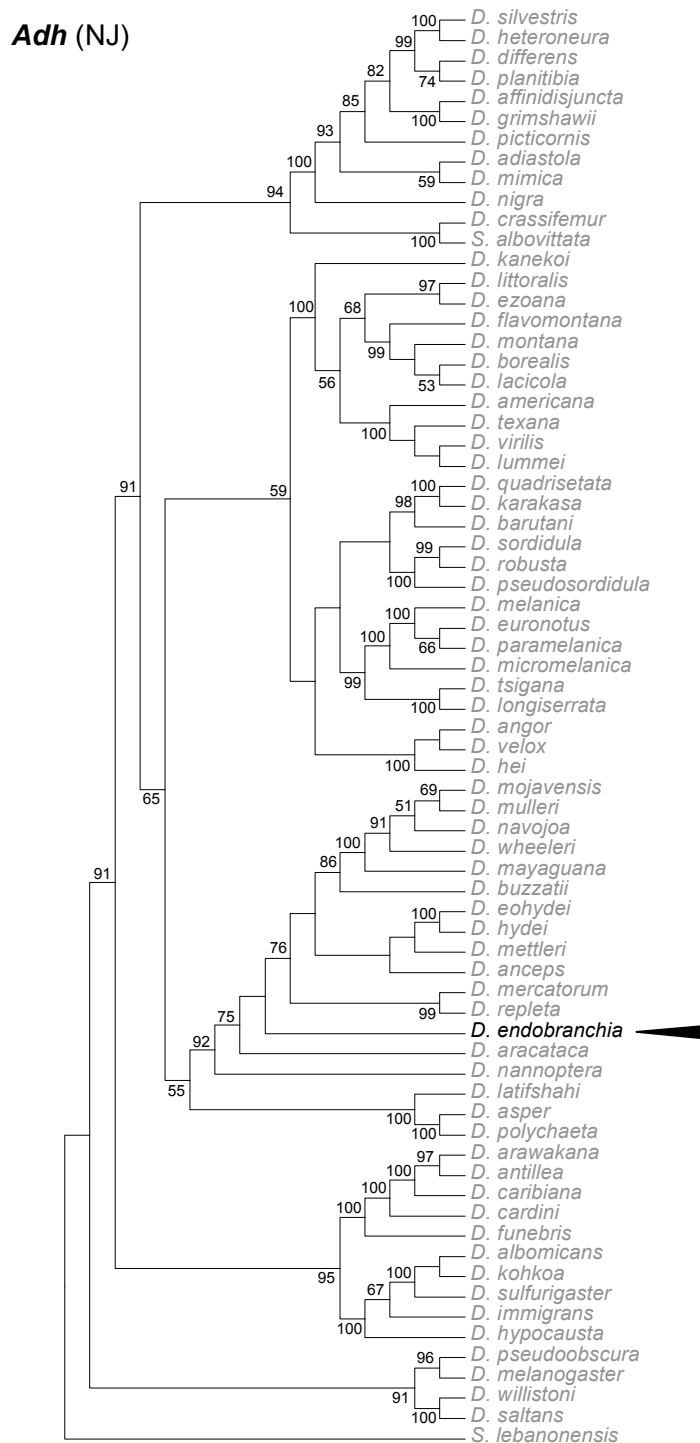

Figure S4 Continues

Figure S4 Continued

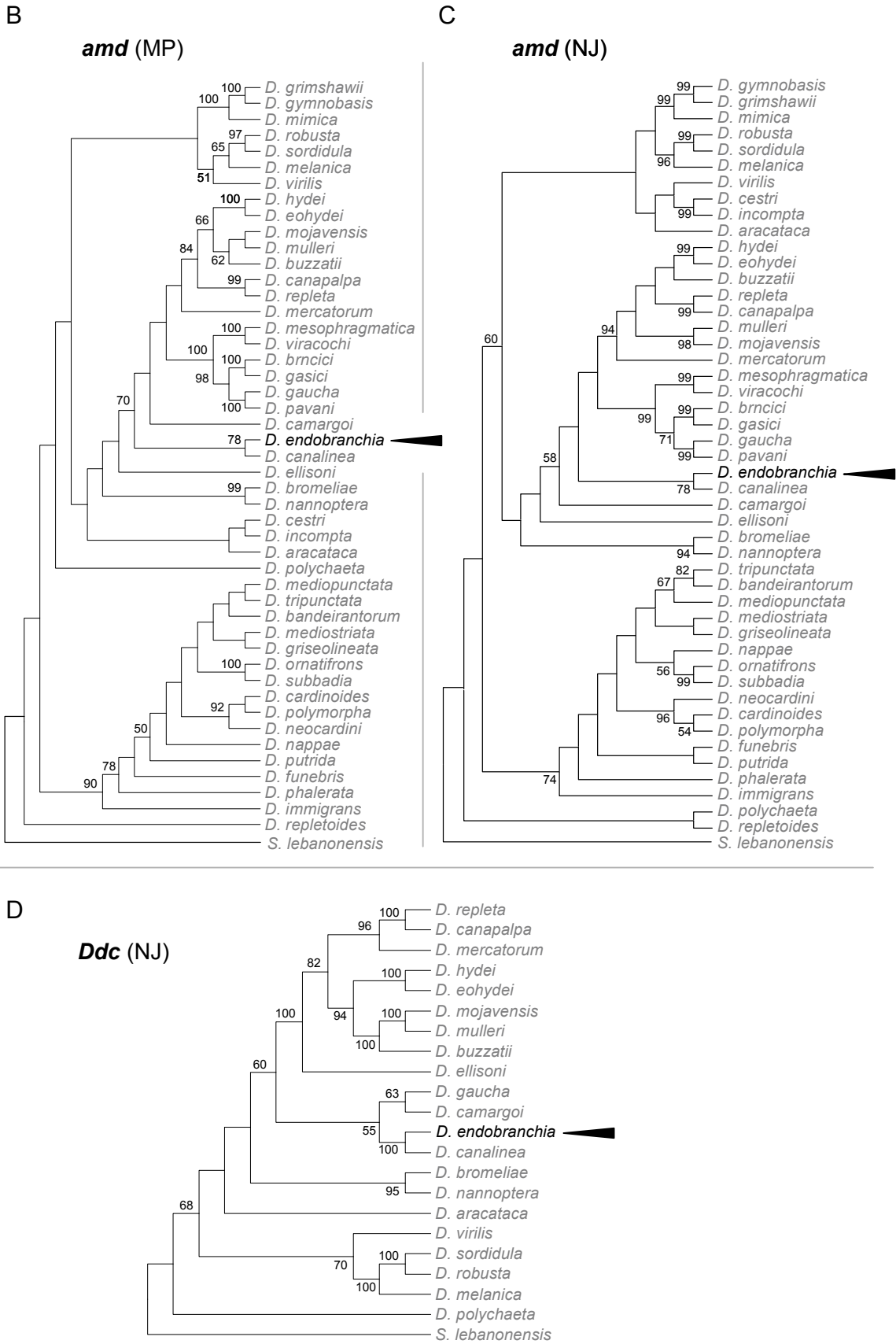

Supplement: Figure S4 — Alternate tree topologies based on Maximum Parsimony (MP) and Neighbor Joining (NJ) analysis of the Adh (A), amd (B, C) and Ddc (D) datasets. Numbers indicate bootstrap support for the corresponding node (1000 replications for NJ, 500 for MP). Values <50% are not shown. (0.62 MB PDF) [file pone.0001942.s005.pdf]

Figure S5

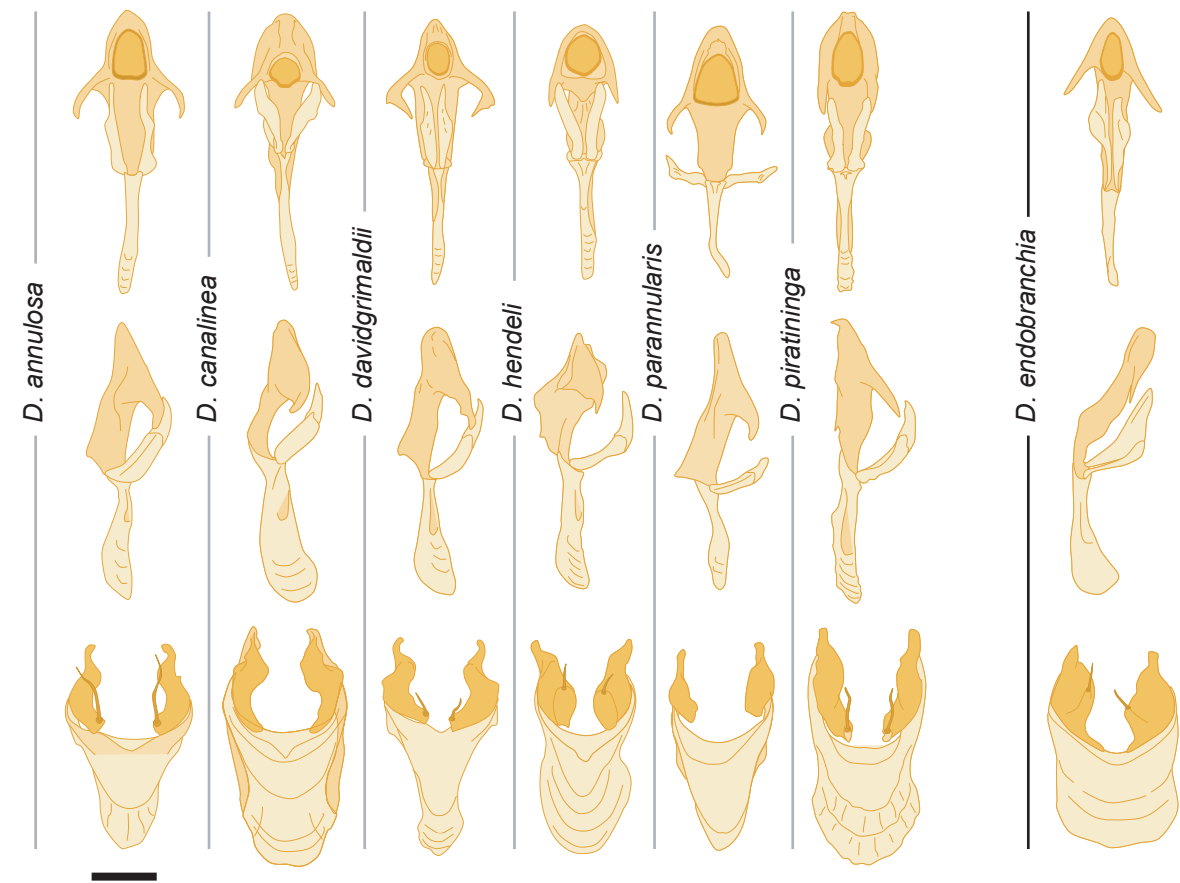

Supplement: Figure S5 — Male internal genitalia from selected members of the canalinea group (redrawn from [13], [26], with kind permission from the publishers) compared with D. endobranchia (right). First and second row, aedeagus (ventral and lateral view respectively); third row, hypandrium (ventral view). Scale bar 0.1 mm. (0.37 MB PDF) [file pone.0001942.s006.pdf]

Figure S6

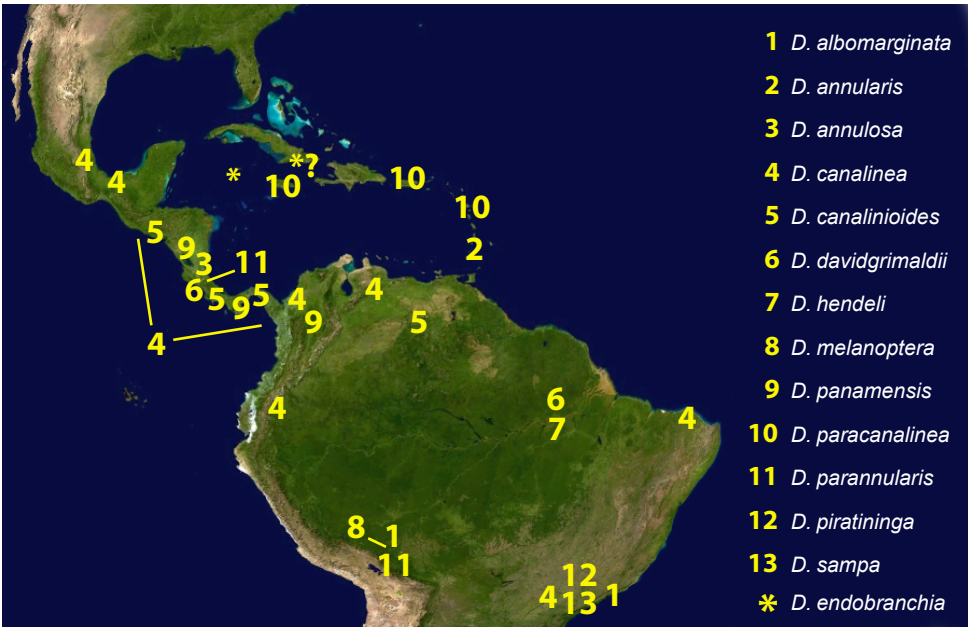

Supplement: Figure S6 — Distribution record of the canalinea group (http://taxodros.unizh.ch and references [12]–[14], [27]–[34]). Satellite image courtesy NASA. (0.40 MB PDF) [file pone.0001942.s007.pdf]
